# Microstructure and Magnetic Field-Induced Strain of a Ni-Mn-Ga-Co-Gd High-Entropy Alloy

**DOI:** 10.3390/ma14102514

**Published:** 2021-05-12

**Authors:** Jia Ju, Liang Hu, Chenwei Bao, Liguo Shuai, Chen Yan, Zhirong Wang

**Affiliations:** 1 Jiangsu Key Laboratory of Advanced Structural Materials and Application Technology, School of Materials Science and Engineering, Nanjing Institute of Technology, Nanjing 211167, China; lianghu0321@126.com; 2 Jiangsu Key Laboratory of Hazardous Chemicals Safety and Control, College of Safety Science and Engineering, Nanjing Tech University, Nanjing 210009, China; 3 Jiangyin Electrical Alloy Limited Liability Company, Wuxi 214423, China; 4 School of Mechanical Engineering, Southeast University, Nanjing 211189, China; 5 College of Mechanics and Materials, Hohai University, Nanjing 211100, China; lovedogw@foxmail.com; 6 Southeast University-China Medical City Institute of Biomedicine and Medical Devices, Taizhou 225300, China; 7 Zhongtian Alloy Technology Co., Ltd., Nantong 226010, China; yanchen@chinaztt.com

**Keywords:** ferromagnetic shape-memory alloy, Ni-Mn-Ga-Co-Gd, high-entropy alloy, martensitic transition, magnetic field-induced strain

## Abstract

The effect of a high-entropy design on martensitic transformation and magnetic field-induced strain has been investigated in the present study for Ni-Mn-Ga-Co-Gd ferromagnetic shape-memory alloys. The purpose was to increase the martensitic transition temperature, as well as the magnetic field-induced strain, of these materials. The results show that there is a co-existence of β, γ, and martensite phases in the microstructure of the alloy samples. Additionally, the martensitic transformation temperature shows a markedly increasing trend for these high-entropy samples, with the largest value being approximately 500 °C. The morphology of the martensite exhibits typical twin characteristics of type L1_0_. Moreover, the magnetic field-induced strain shows an increasing trend, which is caused by the driving force of the twin martensite re-arrangement strengthening.

## 1. Introduction

The ferromagnetic shape-memory alloys (FSMAs) have, during the past few decades, attracted a large amount of interest as potential multifunctional materials for magnetically controlled actuators and sensors. This is mainly due to their large magnetic field-induced strains (MFIS) and fast response times [1,2,3]. For example, the Ni2MnGa-based alloys, with their high recovery strain values (over 10% for a single crystal [4]) and fast response time (less than a millisecond [5]), have found their applications as industrial FSMAs. However, this is not the case for their use in high-frequency actuators and sensors, which is due to their low martensitic transition temperature and high cost of single crystal preparation [6,7,8].

An impressive breakthrough was recently made by Yeh [9], who proposed an entirely new concept of high-entropy alloys. They designed a multielement metallic alloy with a composition close to equimolar (except for the principal element). This metallic alloy was melted into ingots (of about 100 g each) by using an arc furnace and a protection atmosphere. These alloy ingots were, thereafter, undergoing heat treatment for homogenization of the elements. This type of alloy has, when compared with a conventional alloy, a very high mixing entropy, which ensures high phase stability [10,11,12,13]. Their method has later been used for the construction of various types of high-entropy alloys, usually in the form of solid solution, bulk metallic glasses, and intermetallic compounds [14]. Numerous studies have reported that the main phase of a high-entropy alloy is of type B2, to which the structure of the Ni2MnGa-based FSMAs matrix belongs [15,16,17,18,19]. These high-entropy alloys exhibit excellent mechanical and physical properties, which is due to the combination of a high degree of alloying elements in the solution and a sluggish diffusion effect [20]. Lattice distortion in a high-entropy alloy generally leads to increased diffusion activation energies in the lattice, which, in turn, reduces the effective diffusion rate of the atoms. The sluggish diffusion effect is particularly important, since it leads to exceptional high-temperature strengths, impressive high-temperature structural stabilities, and the formation of nanostructures [21]. The slow diffusion, in addition to a resistance against plastic deformations, has been shown to be the perfect condition for a diffusionless martensitic transformation [22]. On the other hand, higher martensitic transformation temperatures have been obtained for high-entropy alloys. This is because the multielement in high-entropy alloy has significant changes in valence electrons per atom and serious effects of atomic size [23,24,25,26].

Polycrystalline high-entropy FSMAs, with four different elemental compositions, have, in the present study, been prepared by using an arc-melting method. The evolution of the microstructures has, then, been investigated, with a focus on the martensite structure and the phase transitions. In addition, the MFIS and the key parameters of the driving force were, for all high-entropy FSMAs, analyzed in detail.

## 2. Materials and Methods

Four polycrystalline FSMA sample ingots were prepared by using high-purity elements (>99.99%, provided by China New Metal Materials Tech. Co., Ltd., Beijing, China) and arc melting (WK-II; provided by Physcience Opto-electronics Co., Ltd., Beijing, China) under an Ar atmosphere. The nominal compositions of the ingots are presented in Table 1. To promote a homogeneous distribution of the elements in the ingots, they were repeatedly flipped and re-melted five times. The ingots were, thereafter, homogenized in vacuum quartz ampoules at 1000 °C for 20 h.

The microstructures of the heat-treated ingots were characterized by a scanning electron microscopy (SEM; FEISirion200, provided by Thermo Fisher, Hillsboro, OR, USA) equipped with an X-ray energy dispersive spectroscopy (EDS; Bruker X-flash detector 4010, provided by Bruker AXS, Karlsruhe, Germany). Moreover, the phase compositions of the ingots were analyzed by using X-ray diffraction (XRD; D8 Advance X, provided by Bruker AXS) with CuKα radiation (λ = 1.5418 Å) in the 2θ range. The XRD spectra were then measured between 10°–90° at room temperature. In addition, the phase structures, and microscopic tissues, were characterized by using transmission electron microscopy (TEM; provided by Tecnai G2 F20, FEI, Hillsboro, Oregon, USA) and selected area electron diffraction (SAED). The TEM test specimens were made thin enough by using a twin jet electro polishing method and a mixed solution of ethanol (95 wt.%) and perchloric acid (5 wt.%). Furthermore, the phase transformation temperatures were characterized by using a differential scanning calorimeter (DSC; STA449 F3, provided by NETZSCH Scientific Instruments Trading Ltd., Netzsch, Selb, Germany). The magnetizations of the samples were examined by using vibrating sample magnetometry (VSM;7407, provided by Lake Shore, Columbus, OH, USA), and the volume fractions of the different phases were determined by means of an image analyzer. The MFISs were, then, measured at a temperature in the range −20 °C to 500 °C, using a metal strain gauge with a magnetic field of 0–7000 Oe.

## 3. Results

### 3.1. Microstructure

**Figure 1:**
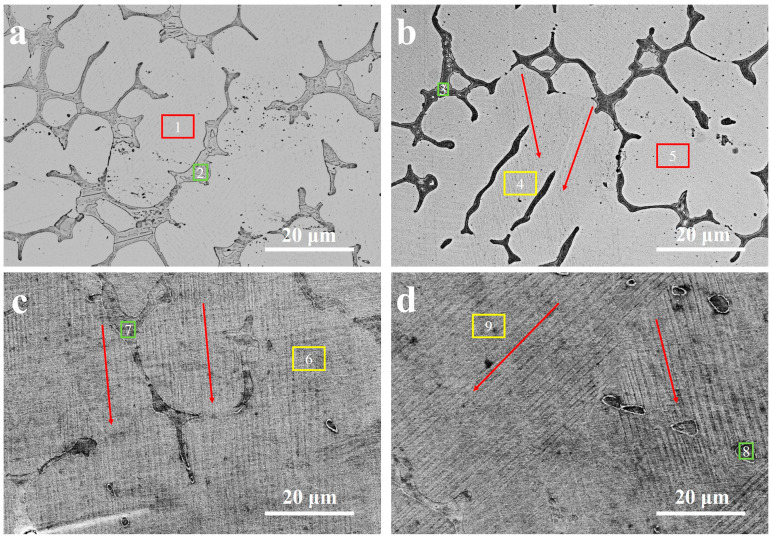
Microstructural SEM characteristics of the alloy ingots; sample (**a**) A1, (**b**) A2, (**c**) A3, and (**d**) A4.

SEM was used for the analysis of the microstructure characteristics of all alloy compositions in the present work (from A1 to A4). As can be seen in Figure 1, multiphase microstructure characteristics were observed in all alloy ingots. A dual-phase structure was found in the A1 sample, with a light-colored matrix phase (red box area 1) and a dark-colored second phase (green box area 2). As can be seen in Figure 1a, the second phase is distributed along the grain boundaries and connects to a dendritic type of formation. The microstructure characteristic of the A2 sample is, however, different (see Figure 1b). In addition to the matrix phase (red box area 5) and the second phase (green box area 3), a lath-type phase (yellow box area 4) was found in the matrix (the red arrows to show the directions of these “laths”). As shown in Figure 1c, the lath-type phase has completely replaced the matrix phase in sample A3 (yellow box area 6). The directions of these “laths” are shown by red arrows in the figure. In addition, the second phase (green box area 7) has gradually decreased and is no longer continuously distributed along the grain boundaries. Moreover, the microstructure characteristics of sample A4 are still of a dual-phase type, with a lath-type phase (yellow box area 9) and a second phase (green box area 8) (see Figure 1d). The directions of the “laths” are demonstrated by red arrows. Additionally, the second phase has continued to decrease, and the distribution is more scattered at the grain boundaries.

The chemical compositions of the different phases in the alloy samples (A1–A4) have been characterized using EDS. As can be seen in Table 2, it is clear that the compositions are quite different for samples A1 to A4. The composition of the matrix is almost equimolar for sample A1; Ni:Mn:Ga:Co ≈ 1:1:1:1). In comparison, the approximate atomic ratios in the second phase of A1 are Ni:Mn:Ga:Co ≈ 1.1:1:1.9:2.5, which indicates that the Ga and Co elements have aggregated in this second phase. For alloy A2, the lath-type phase, which is positioned in the matrix phase, has the same composition as the matrix phase; Ni:Mn:Ga:Co ≈ 1:1:1:1. Hence, there is no need for an atomic diffusion in the formation of this lath-type phase. Moreover, the composition of the second phase shows a similar picture as was the case for the A1 alloy. The atomic ratios are Ni:Mn:Ga:Gd ≈ 1:1:2.5:2.6, indicating segregation of the Ga and Gd elements. Furthermore, the chemical composition of the lath-type phase is also Ni:Mn:Ga:Co ≈ 1:1:1:1 for the A3 and A4 alloys. In addition, the approximate atomic ratios in the second phases are Ni:Mn:Ga:Co:Gd ≈ 1.7:1:1:2.6:1.5 and Ni:Mn:Ga:Co:Gd ≈ 1:1.1:1:2.9:2.4, respectively. As was the situation with the A1 and A2 alloys, the elements Co and Gd have undergone segregation in this second phase. In addition, this is also the situation for Ni in the A3 alloy.

**Figure 2:**
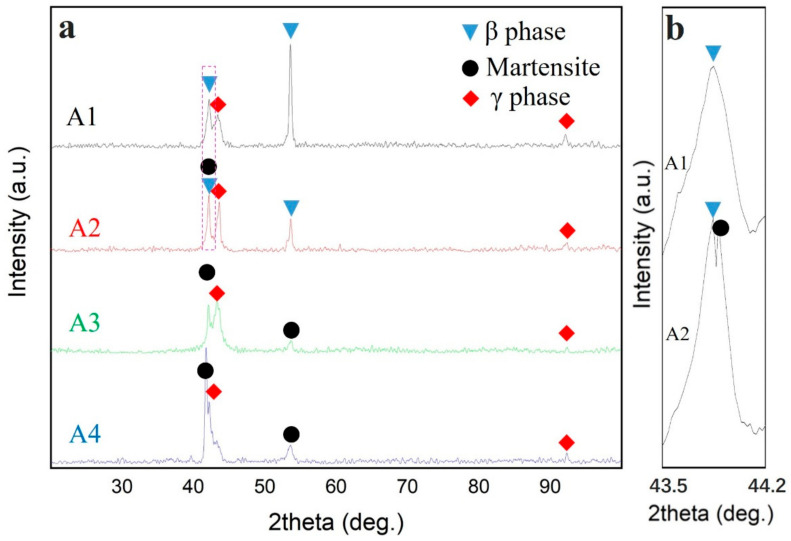
XRD patterns of the alloy samples A1 to A4 (**a**) and a partial enlargement of the red box line area in XRD patterns (**b**).

The XRD patterns of the alloy samples (A1–A4) at room temperature are shown in Figure 2. These results are consistent with the SEM observations and EDS analyses. The major peaks of A1 are identified as the β phase (B2 type) and γ phase (A1 type) of austenite. [These austenite phases are, from here-on, called the β phase and γ phase.] More specifically, the matrix and the second phase in A1 consist of the β phase and γ phase, respectively. However, for the A2 sample, the diffraction peaks correspond to martensite (L1_0_ type) in addition to the β phase (B2 type) and γ phase (A1 type). Some peaks, that can be indexed as L1_0_-type martensite, suggest that the austenite undergoes martensitic transformation from B2-type austenite to L1_0_-type martensite. Moreover, the diffraction peaks for the A3 alloy sample show that the β phase has been completely replaced by martensite, meaning that the martensitic transformation temperature is over room temperature. Hence, the martensitic transformation process has, in A3, become completely finished, which was not the case for the A2 sample. Moreover, the peaks of the γ phase at 44.8° were very weak, indicating that the content of the γ phase, in the A3 alloy, was largely reduced. Finally, for the A4 sample, the diffraction peaks of martensite at 43.9° were stronger, while the peaks of the γ phase at 43.1° were weaker, in comparison to A1 to A3.

### 3.2. Martensitic Transition

**Figure 3:**
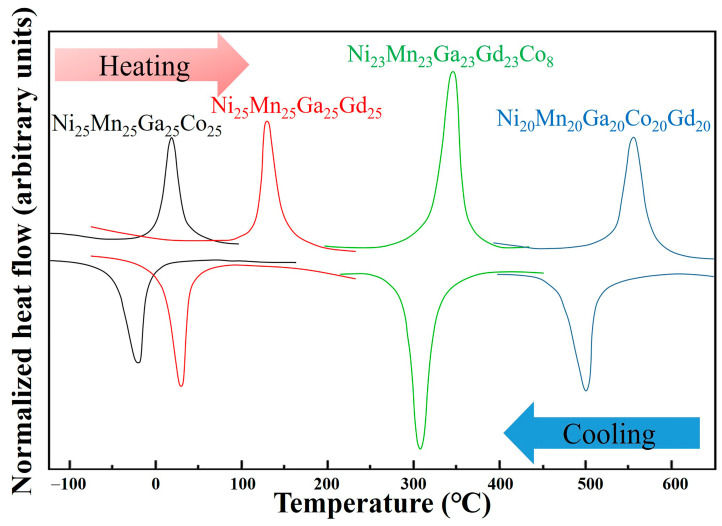
DSC curves obtained during thermal cycling in the region −120 °C to 650 °C: Black: A1; Red: A2; Green: A3; Blue: A4.

DSC curves, obtained for the alloy samples during thermal cycling in the temperature region −120°C to 650 °C, are shown in Figure 3. It is obvious that an endothermic and exothermic peak will occur when heating and cooling the sample, respectively. This is a strong indication of the fact that a reversible martensite transition will occur during thermal cycling. The martensitic transformation temperatures, including martensite start (Ms) and final martensite (Mf), as well as the austenite start (As) and final austenite (Af), can be determined from the DSC curves in Figure 3. They are, in addition, listed in Table 3. As can be seen in Figure 3 and Table 3, there is a clear trend in martensitic transformation temperatures when going from A1 to A4. The Ms of sample A1 is just 6 °C, which indicates that martensite cannot be found at room temperature in this specific alloy (as shown in Figure 1a). For the A2 sample, the Mf is 25 °C, which suggests that martensite and austenite will coexist at room temperature (also supported by the results presented in Figure 1b). In addition, the Mf of each of the A3 and A4 samples is well-above room temperature, indicating that the martensite has completely replaced austenite (as supported by Figure 1b,d).

**Figure 4:**
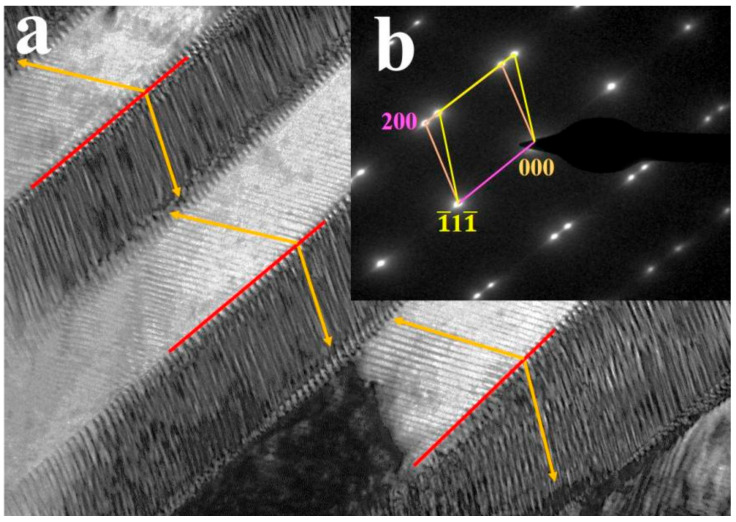
Martensite morphology (**a**) and structure (**b**) obtained by TEM and SAED, respectively.

TEM and SAED characterizations were, furthermore, used in analyzing the morphology and structure of martensite in A4 (see Figure 4). As can be seen in this figure, it is obvious that the martensite morphology, which consists of alternating black and white parallel stripes, has a typical twin martensitic characteristic (Figure 4a). Besides, the SAED of the martensite (along the [001] zone axis) confirms that the diffraction spots belong to the L1_0_ twin structure, with a (111) twinning plane (see Figure 4b).

### 3.3. Magnetic Field-Induced Strain

**Figure 5:**
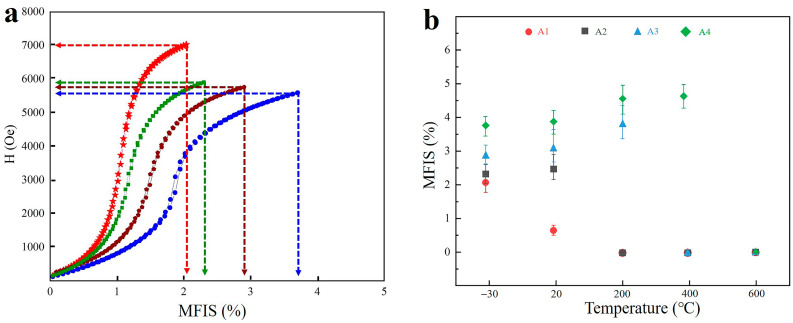
The MFISs of alloy samples under different types of driving fields; (**a**) magnetic field, and (**b**) thermal fields.

Figure 5 shows the MFISs under different types of driving fields (magnetic field and magnetic + thermal fields). As presented in Figure 5a, the sample ingots show an apparent output strain, and slight strain lag, during the increase, and decrease, of the magnetic field, respectively. The maximum strain of A1 is 2.05% at a magnetic field of 7000 Oe (at room temperature). Furthermore, the maximum strain is 2.31%, at 5890 Oe, for the A2 sample, and the maximum strain is even larger for A3 (2.92% at 5728 Oe). Finally, the maximum strain of A4 is 3.79% at 4996 Oe. The evolution of MFIS, in varying the temperature (starting form 600 °C cooling to −30 °C) at a fixed magnetic field (5000 Oe), was also studied, and the results can be seen in Figure 5b. For lower temperatures (−30 °C), the MFIS trend is identical to the one shown in Figure 5a (i.e., the maximum strain will increase when going from A1 to A4). At a slightly larger temperature (20 °C), the MFISs of A1, A2, and A4 show just a slightly increasing trend. However, the A1 sample is an exception, for which the MFIS decreases from 2.05 % to 0.57%. The underlying reasons for this observation may be the occurrence of an austenitic transformation and/or that a part of the twin martensite structure has disappeared at the temperature of 20 °C. Moreover, when the temperature increases to 200 °C, the MFISs of A3 and A4 show a significantly increasing trend (3.72% and 4.51%, respectively). In contrast, no ferromagnetic shape-memory effect could be found for the A2 and A3 samples. This is most probably due to the complete transformation of austenite at this very high temperature. As the temperature was further increased (to 400 °C), it was only the A4 sample that showed an increasing trend in MFIS (but only a minor one). The disappearance of a ferromagnetic shape-memory effect for the A1, A2, and A3 samples can be explained by the disappearance of the twin martensite phases in these materials. When increasing the temperature to 600 °C, no twin martensite structure could be detected in any of the A1 to A4 samples, and with undetectable MFISs in the magnetic field.

## 4. Discussion

### 4.1. Microstructure Evolution and Martensite Transition

**Figure 6:**
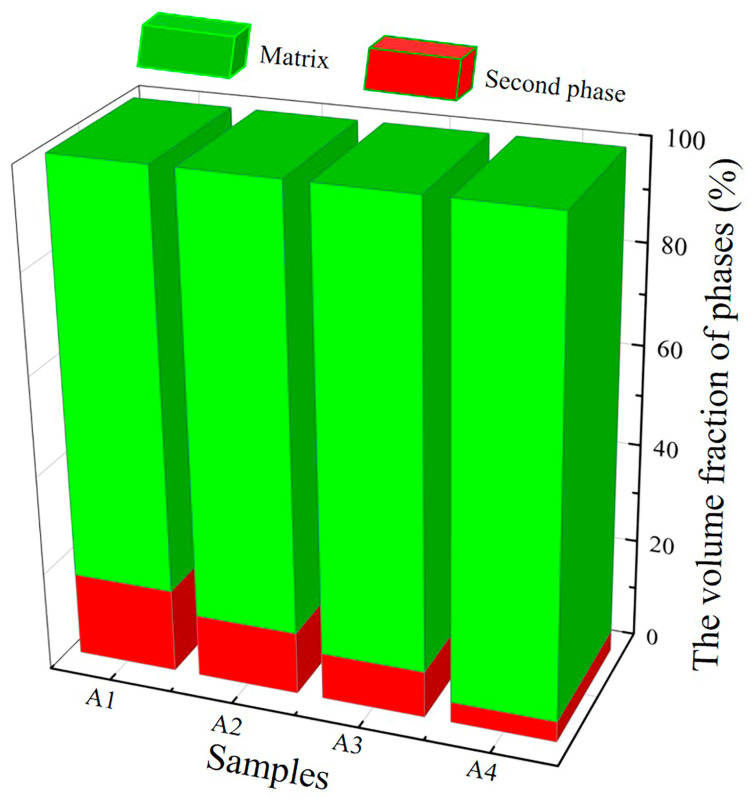
Volume fractions of the second phase and matrix in the various alloy samples (A1–A4).

As a result of the microstructure analysis, two main variations were observed for the A1 to A4 alloy samples. Firstly, the contents of the second phase (γ phase) and the matrix (β phase or martensite) differ markedly for the studied samples. Figure 6 shows the volume fraction of the second phase and matrix in each ingot sample. As was also shown in Figure 1, the volume fraction of the γ phase decreases from 11.37% to 2.67%, when going from A1 to A4. As is generally known, a multielement alloy with a close-to-equimolar composition, and with the entropy of mixing being larger than the ones for the existing alloy and the solid solutions of the alloying elements, is a microstructural characteristic of a high-entropy alloy [27,28,29]. In the present study, the reason for the decreased content of γ phase can be ascribed the high-entropy design of the alloys (i.e., by using a solution containing many alloying elements). The other main variation amongst the A1 to A4 samples is that martensite gradually replaces the matrix in the samples (as observed by SEM). This is consistent with the martensitic transformation temperatures (as analyzed by DSC).

For FSMAs, the martensitic transformation is extremely sensitive to the change in alloy composition. This is because the valence electron concentration (Re/a), in the alloy, is linearly correlated with the martensitic transformation temperature [30,31]. In the present study, and for the high-entropy design of alloys, the relation between Ms and Re/a can be determined by using Equation (1) [32]:

(1) $$\frac{M_{s}}{10,000}=-1.478+0.1933\times R_{\frac{e}{a}}-0.1643\times \left(S^{↑}-S^{↓}\right)$$

where *S*↑ and *S*↓ are the spin-up and spin-down electrons, respectively. Moreover, Re/a can be obtained by using Equation (2) [33]:

(2) Re/a=∑(Ve×Pe)

where Ve is the number of atomic valence electrons and Pe is the proportion of the element e.

**Figure 7:**
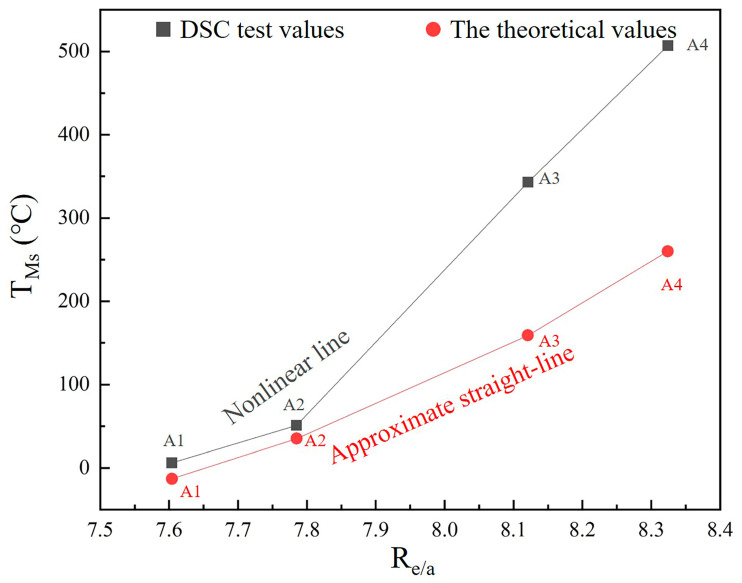
The relation between Re/a and Ms in the alloys.

According to previous studies, there is a linear correlation between the Re/a values and Ms for the FSMAs [24,25]. For all alloy samples in the present study, the calculated Re/a values, together with the relation between Re/a and Ms, are shown in Figure 7. It is worth noting that the calculated Ms values are generally smaller than the experimentally obtained ones by using DSC analysis. However, the calculated values are obtained from a linear correlation between the Re/values, while the experimental ones are obtained without any linear correlation. The reason for this discrepancy is most probably the large size-dependency of the high-entropy design of the alloys. In the present study, the high-entropy alloys were designed by using five elements with difference atomic radii (as listed in Table 2). Thus, a large variation in atomic size is expected to cause large lattice distortion in the samples. Moreover, the volume of the unit cell model must be increased, since many elements are used in the model. This circumstance results in a change in the relative positions of the Brillouin zone boundaries and Fermi surfaces.

### 4.2. MFIS Variations

Earlier research has indicated that the MFIS of FSMAs depends on the driving force that is coupled to the rearrangement of the twin boundaries [34,35]. The driving force can be expressed by using a dimensionless field parameter (Df) (see Equation (3)) [36]:

(3) $$\mathrm{Df}=\frac{\mathrm{Ms}\cdot H}{2K_{u}}$$

where Ms·H is the Zeeman energy and Ku are the magneto crystalline anisotropy energy. Moreover, Ms is the saturation magnetization and H is the applied magnetic field. The Ms·H and *Ku* terms are the resistance and acceleration, respectively, of the twin martensite rearrangement. Hence, the acceleration is larger than the resistance for a Df value less than one, and a macroscopic strain will occur due to the variations in the twin martensite rearrangement [36].

**Figure 8:**
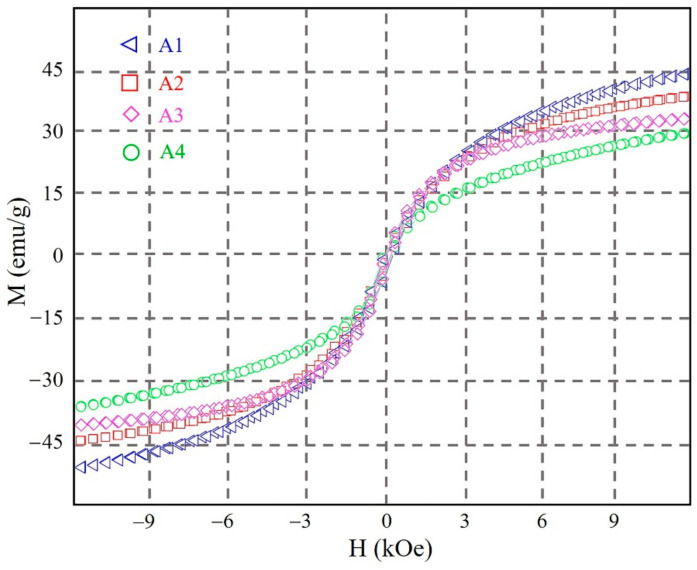
The magnetization vs. applied field magnetic hysteresis loop curves for the alloy samples A1 to A4.

The key parameters (Ms and Ku) of the driving force have been analyzed in the present study. Figure 8 shows the magnetization vs. applied field magnetic hysteresis loop curves for the different alloy samples (obtained by VSM). As can be seen in Figure 8, the Ms values of the alloys show a clear decreasing trend with a change in alloy composition. The Ms values are 44.15876, 40.28641, 32.01486, and 28.79316 emu/g for A1, A2, A3, and A4, respectively. With reference to the results obtained in previous studies, the decrease in Ms will significantly weaken the resistance of the martensite twins against re-migration. Hence, the resistance of the MFISs also shows a decreasing trend for the alloy samples in the present study.

**Figure 9:**
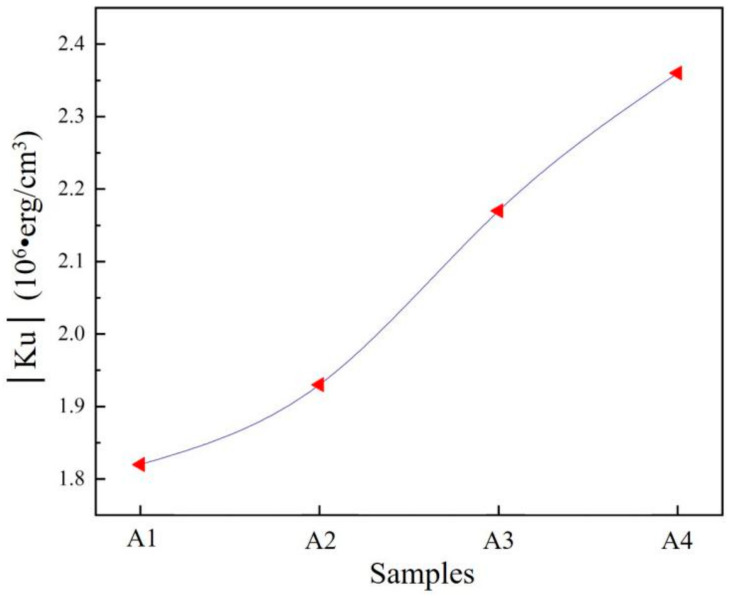
Ku values for the alloy samples A1 to A4.

To secure the twin martensite rearrangements in the A1 to A4 alloy samples, another key parameter, *Ku*, has here been calculated. These calculations are based on the Sucksmith-Thompson method (see Equations (4) and (5)) [36].

(4) $$\;2\times \frac{K_{2}^{\prime }}{M_{s}^{2}}+4\times \frac{K_{4}^{\prime }}{M_{s}^{4}}\times M2=\frac{H_{e}}{M}$$

(5) $$Ku\approx K_{2}^{\prime }+K_{4}^{\prime }$$

where *M* is the magnetization, *He* is the effective field, $K_{2}^{\prime }$ is the second magnetocrystalline anisotropy constant, and $K_{4}^{\prime }$ is the fourth-order magnetocrystalline anisotropy constant. Figure 9 shows the Ku values for the alloy samples in the present study. These Ku values show a clear increasing trend, from 1.82 × 10^6^ · erg/cm^3^ to 2.36 × 10^6^ · erg/cm^3^, when going from A1 to A4. Based on these results, the acceleration of the twin martensite rearrangement can be enhanced as the Ku values increase. This might be due to the strengthened pinning effect when using a larger number of alloying elements. In the high-entropy design, a large driving force has, therefore, been obtained, which is due to the acceleration of the increased twin martensite rearrangement and the reduced resistance [28].

## 5. Conclusions

The microstructures and ferromagnetic shape memories, of four high-entropy Ni-Mn-Ga-Co-Gd FSMAs have, in the present study, been analyzed and the main conclusions are listed below:

(1) The microstructures of the alloy samples are a mixture of multiphases, including the β phase + γ phase in alloy sample A1, β phase + martensite + γ phase in alloy sample A2, and martensite + γ phase in alloy samples A3 and A4.
(2) There is a clear trend in martensitic transformation temperatures for the various alloy samples. The transformation temperature increases when going from A1 to A4 with an increase in Re/a. For example, the Ms values of A3 and A4 are higher than 300 °C, and this increases the possibility for the high-entropy Ni-Mn-Ga-Co-Gd FSMAs to be used for high-temperature applications.
(3) The martensite morphologies of the alloy samples exhibit typical twin martensite characteristics, with a martensitic structure of type L1_0_ (forming twin grains in the (111) twinning plane).
(4) The MFIS of the studied alloys show an increasing trend from A1 to A4. This is due to the driving force of the twin martensite rearrangement strengthening. In the high-entropy design of the Ni-Mn-Ga-Co-Gd FSMAs, the Ms of the alloys, being a key parameter for the resistance against twin martensite rearrangements, will gradually decrease. Conversely, the Ku value of the alloy, as a key parameter of the acceleration of the twin martensite rearrangement, will gradually increase.

## Figures and Tables

**Table 1:** Nominal compositions of the alloy ingots.

Alloy Ingot	Nominal Compositions (at. %)				
	Ni	Mn	Ga	Co	Gd
A1	25	25	25	25	/
A2	25	25	25	/	25
A3	34	20	20	20	6
A4	20	20	20	20	20

**Table 2:** EDS analysis of the chemicals’ compositions in the different phases of A1 to A4.

Alloy Ingot	Phase	Scan Area in the SEM Image	Actual Composition by EDS Analysis (at. %)					Approximate Atomic Ratio
			Ni	Mn	Ga	Co	Gd	
A1	Matrix	Red box area 1	25.7	25.2	24.5	24.6	/	1:1:1:1
	Second phase	Green box area 2	16.1	15.3	29.7	38.9	/	1.1:1:1.9:2.5
A2	Matrix	Red box area 5	25.4	24.8	25.6	/	24.2	1:1:1:1
	Lath-type phase	Yellow box area 4	25.6	25.1	24.7	/	24.6	1:1:1:1
	Second phase	Green box area 3	13.9	14.4	35.2	/	36.5	1:1:2.5:2.6
A3	Lath-type phase	Yellow box area 6	35.1	19.4	19.8	21.5	5.2	6.8:3.7:3.8:4.1:1
	Second phase	Green box area 7	21.7	13.2	12.8	33.4	18.9	1.7:1:1:2.6:1.5
A4	Lath-type phase	Yellow box area 9	20.6	20.9	18.8	20.3	19.2	1:1:1:1
	Second phase	Green box area 8	12.3	12.5	11.9	34.4	28.9	1:1.1:1:2.9:2.4

**Table 3:** Representative transformation temperatures for the alloy samples A1 to A4.

Alloy Ingot	Martensitic Transformation Temperature (°C)			
	Ms	Mf	As	Af
A1	6	−28	4	47
A2	51	25	126	161
A3	343	288	321	369
A4	507	482	539	568

## Data Availability

The data presented in this study are available on request from the corresponding author.
